# SBR Vulcanizates Filled with Modified Ground Tire Rubber

**DOI:** 10.3390/ma14143991

**Published:** 2021-07-16

**Authors:** Katarzyna Klajn, Tomasz Gozdek, Dariusz M. Bieliński, Mariusz Siciński, Magdalena Zarzecka-Napierała, Zbigniew Pędzich

**Affiliations:** 1 Institute of Polymer & Dye Technology, Lodz University of Technology, Stefanowskiego 16, 90-537 Lodz, Poland; katarzyna.klajn@dokt.p.lodz.pl (K.K.); mariusz.sicinski@p.lodz.pl (M.S.); 2 Department of Ceramics and Refractories, AGH University of Science and Technology, Mickiewicza 30, 30-059 Cracow, Poland; zarzecka@agh.edu.pl (M.Z.-N.); pedzich@agh.edu.pl (Z.P.)

**Keywords:** GTR, surface modification, acid activation, silanization, rubber vulcanizates, mechanical properties, tribological properties

## Abstract

Ground tire rubber (GTR) is used to decrease the cost of vulcanizates. However, insufficient interactions between GTR particles and rubber matrices make mechanical properties of vulcanizates containing GTR deteriorate. This paper compares original methods of GTR modification. The effects of surface activation of GTR by sulfuric acid (A), its modification by (3-mercaptopropyl)trimethoxy silane (M), or the hybrid treatment—combining both approaches (H), were analyzed in terms of surface energy, specific surface area and morphology of GTR particles. Vulcanizates containing virgin GTR were compared to the rubber filled with the modified GTR particles keeping the same amount of CB in the rubber mix, according to their crosslink density, mechanical and tribological properties. Contrary to the virgin GTR, the addition of modified GTR increases the stiffness of the vulcanizates. The highest changes have been observed for the samples filled with ca. 12 phr of the GTR modified with silane and ca. 25 phr of the GTR subjected to the hybrid treatment, representing the highest crosslink density of rubber vulcanizates filled with GTR. Furthermore, the addition of modified GTR, especially in the case of the samples where 10 phr of rubber was replaced, results in the significant lowering of friction but higher abrasive wear.

## 1. Introduction

The continuous development of the car industry is a reason for the increasing amount of waste from end-of-life vehicles and their components. Each car has about 60 kg of rubber parts, of which 2/3 originates from tires [1]. In 2018, member countries of the European Union “generated” ca. 3.4 million tons of used tires, of which about 1/3 were stored [2]. Recycling of this type of waste is problematic, due to their different and complex composition. However, there are several options to reuse worn car tires, such as retreading, burning to produce energy, or material recycling. The biggest potential for application in rubber technology, due to simplicity and economy, seems to be ground tire rubber (GTR). The optimal fraction size of GTR, suitable to use as a filler in rubber mixes, is a powder with an average particle size below 1 mm [3].

GTR has the lowest size among other recycling products, e.g., chips (10–50 mm) or granulates (1–10 mm) [4]. However, in comparison to conventional reinforcing fillers, such as silica or carbon black, GTR particles are many times bigger in size. Because of that, attempts to introduce a GTR powder into the rubber matrix, instead of conventional reinforcing fillers, adversely affect the mechanical properties of the rubber vulcanizates. However, it can still be used as a partial replacement of active fillers or secondary fillers when high mechanical properties of products in the case of, for example, car mats, shoe soles, mats for animals, roof or floor coverings, etc. are not required [1,5,6].

Due to the different morphology and, to some extent, the chemical composition of GTR particles’ surface resulting from the method of their grinding [4,6], it is important not only to characterize the phase composition, size, and shape of the particles, but also their surface according to its topography, specific surface area, surface energy and overall chemical activity [7,8,9]. Usually, the first step is to decide GTR loading, to some extent determined by the mechanical properties required by the application. The use of GTR as a filler can significantly increase the degree of replacing virgin material with recycled material [10]. Unfortunately, due to weak interactions between GTR and the polymer matrix, the addition of pure GTR is limited by a negative effect on the mechanical properties of rubber [11]. Nevertheless, Carli et al. applied ground tire rubber directly as a filler [12,13]. Another approach has been presented by Yehia et al., who used GTR to replace part of carbon black [14]. Undoubtedly, the compatibilization of GTR with the rubber matrix is enhanced when subjecting waste rubber to thermomechanical processes, as described by Kolinski et al. [15].

Despite optimization of the composition, the problem with interactions between ground tire rubber and polymer matrices still remains unsolved and attracts attention. Modification of the GTR’s surface creates a possibility to improve the compatibility between GTR particles and the polymer matrix. It can be made by physical methods, such as microwaves [16] or low-temperature plasma treatment [17], but the most common approach, due to its cost effectiveness, is chemical modification of GTR’s surface. Therefore, the most popular methods so far remain oxidation realized by sulfuric acid, nitric acid, trichloroisocyanuric acid (TCI), or hydrogen peroxide treatment [10,14,17]. Better compatibilization observed could be explained by the affinity of sulfur which is polar in nature, and other components of crosslinking systems, resulting in their adsorption on the surface of GTR particles, similarly to particles of active fillers [18]. Despite the oxidation significantly increasing the polarity of GTR’s surface, the modifications are very often accompanied by the cracking of the surface layer, leading to the development of surface geometry of GTR particles, which facilitates their penetration by polymer macromolecules. It happens that, even for nonpolar polymer matrices, such physical interactions take over the effects of chemical modifications [14,19]. Another way of improving the phase compatibility is using adequate coupling agents, which can attach to chemical groups reactive towards rubber, present on the surface of GTR particles [10]. Commonly used coupling agents are silanes or maleic anhydride grafted polymers [10,20,21], but the application of low molecular weight polymers or oligomers has also been reported [22]. The maleination of polymers promotes their adhesion to several substrates [23]. Other functionalizations involving epoxidized, carboxylated, hydroxyl terminated or methacrylated macromolecules are also used in this sense to provide extra adhesion to polar surfaces, including metallic or glassy surfaces. Despite the numerous attempts made so far, the optimization of the interactions still requires extensive research on appropriate modifiers, according to their quantity and application sequence.

In this work, an original approach to the problem of compatibilization between GTR particles and the rubber matrix is presented. Ground Tire Rubber after oxidative activation using sulfuric acid was subjected to treatment with (3-mercaptopropyl)trimethoxysilane. Such a particular hybrid treatment has never been used before; however, surface activation of GTR particles prior to their final treatment is known and already reported in the subject literature [10,20,24].

## 2. Experimental

### 2.1. Methods of GTR Powder Modification

#### 2.1.1. Oxidative Treatment

Surface activation of GTR powder—sample A (Orzeł S.A., Poniatowa, Poland) was performed by placing 100 g of a powder in an ice-bathed flask; next, 400 mL of 96 % sulfuric acid (Chempur, Piekary Śląskie, Poland) was dropped in and the whole flask was stirred for 10 min. After that, the powder was vacuum filtrated and rinsed extensively with 15 wt. % ammonia (Chempur, Piekary Śląskie, Poland) solution and deionized hot water to obtain a neutral pH of the GTR surface.

#### 2.1.2. Silane Treatment

Surface modification of GTR powder (sample M) was carried out by placing 100 g of a powder in a flask containing 400 mL of methanol and 2 mL of (3-mercaptopropyl) trimethoxysilane (Aldrich, saimt Louis, MO, USA), which was subsequently subjected to heating at 70 °C to evaporate the solvent. After that, the powder was vacuum filtrated and rinsed extensively with deionized water.

#### 2.1.3. Hybrid Treatment

Hybrid modification of GTR powder (sample H) was a combination of the methods described above. Activation with sulfuric acid was followed by the treatment of the dried powder particles with the methanol solution of silane. The oxidized surface of the GTR was expected to exhibit greater chemical reactivity towards the methoxy groups of the silane [25]. Before application in rubber mixes, all types of GTR powders were dried to a constant mass at 70 °C.

The yield of the chemical treatments described in Section 2.1.1, Section 2.1.2 and Section 2.1.3 were verified by determination of the changes to chemical composition of the GTR (FTIR, EDS), surface polarity, specific surface area and surface morphology (SEM and optical microscopy) of the particles (see Section 2.3).

### 2.2. Preparation of Rubber Mixes

The previously applied approach, taking into account the carbon black and rubber content in the GTR powder, when calculating the composition of the rubber mixes studied [26], seems to be more accurate from the point of view of the influence of filler content (already present in GTR particles) on the mechanical properties of the rubber vulcanizates. GTR powder was provided by Orzeł S.A. (Orzel Powder 0–0.8 mm, Poniatowa, Poland). Characteristics of the material prior to its modification were performed and the results are presented in the next chapter.

The composition of SBR (Ker 1500, Synthos S.A., Poland)-based rubber mixes was modified by the introduction of various amounts of different GTRs (virgin: UN or modified: A, M, or H) instead of a part of the rubber. The amount of GTR was adjusted individually (based on TGA data) to compensate 5 or 10 wt. parts of SBR by the rubber contained in the GTR—Table 1. The amount of carbon black in the rubber mixes was also reduced adequately to the filler content in the GTR added, determined by TG analysis (Table 3). In this way, the total amounts of rubber and carbon black in the mixes were kept constant. All ingredients were mixed in a Brabender Plasticorder (Germany) laboratory 80 cm^3^ mixer in three steps, according to the PN-ISO 2393:2015-12 standard (1st: SBR, carbon black and stearic acid, 2nd: mixture from the first step, GTR and zinc oxide, 3rd: mixture from the second step, CBS and sulfur; time of each step: 5 min, temperature up to 60 °C), and sheeted with a David Bridge (UK) laboratory two-rolls mill. A rubber mix of SBR filled with CB (not containing any GTR) was made for comparison.

### 2.3. Characterization of GTR

#### 2.3.1. Particle Size Distribution

Size distribution analysis of GTR samples was performed by sieve analysis using a sieve shaker AS 200 control (Retsch, Haan, Germany), operating with 4, 2, 1, 0.5, 0.25, 0.125, 0.063 and 0.045 mm sieves. Experimental conditions applied: test time—3 min, amplitude 1.5 mm. The measurement error was calculated using the student’s t-test for α = 0.005.

#### 2.3.2. Particle Shape

The shape of GTR particles was studied with an optical microscope Optatech (Warsaw, Poland), operating under a magnification of 64 or 100 times, combined with a Leica MZ 6 (Wetzlar, Germany) camera and OptaView (Optatech, Warsaw, Poland) software. GTR particles were examined as received, without any prior preparation.

#### 2.3.3. Surface Polarity

Surface free energy (SFE) was determined by a KRÜSS tensiometer (Hamburg, Germany). The capillary constant was determined with heptane. Other solvents applied to designate SFE were 1,4-dioxane and methanol. The used method: Owens–Wendt–Rabel–Kaelble (OWRK) [27]. Due to the too-big size of the GTR particles, the method could only be applied for the comparison of the trends represented by changes (without comparison of values) to surface polarity. This is why the experimental error was not analyzed this time.

#### 2.3.4. Chemical Activity

Fourier transform infrared spectroscopy (FTIR) absorbance spectra of GTR surfaces were collected in the 4000–400 cm^−1^ range (64 scans, resolution of 4 cm^−1^). Experiments were performed with a Nicolet 6700 FTIR spectrometer equipped with a Smart Orbit ATR sampling accessory (both from Thermo Scientific, Waltham, MA, USA), operating with a diamond crystal.

#### 2.3.5. Specific Surface Area

The specific surface area (SSA) of GTR powders was determined with an ASAP 2010 (Micromeritics, Norcross, GA, USA) instrument, applying the Brunauer–Emmet–Teller (BET) equation. The specific surface area was determined utilising the 5-point BET procedure, according to ASTM D3037 and ASTM D4820 standards. The measurement error was calculated using the student’s t-test for α = 0.005.

#### 2.3.6. Morphology and Composition of the Surface

Changes in the morphology of GTR particles subjected to surface modification were studied with a Nova Nano SEM 200 FEI (Thermo Scientific, Hilsboro, OR, USA) equipped with an EDS analyzer and a BSE detector, operating under low vacuum conditions. Observations were performed on the surfaces covered by a graphite layer for improving the sample conductivity. The voltage used during observations was experimentally fixed to 1200 kV, which assured the proper quality of images. A low vacuum detector (LVD) was used. The tilt was stable and fixed at 0°. A used magnification of 500× allowed us to observe differences between samples.

#### 2.3.7. Composition

**Figure 1:**
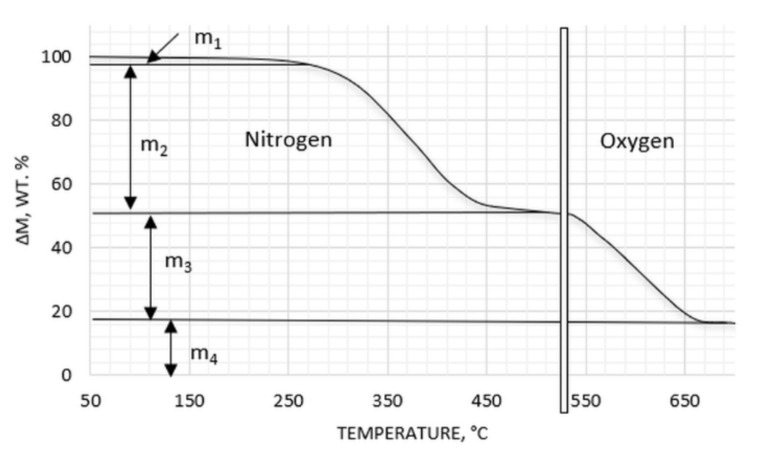
TGA analysis procedure used for the analysis of GTR samples. m_1_—low molecular weight organic substances and water, m_2_—rubber, m_3_—carbon black, m_4_—mineral residue.

The fundamental composition of GTR samples was determined thermogravimetrically, using a Netzsch TG 209 (Selb, Germany) instrument. A two-step procedure was applied: 1st step: heating from 30 °C up to 550 °C under a nitrogen flow of 16 mL/min, with a heating rate of 10 °C/min, and 2nd step: heating from 550 °C to 830 °C under an oxygen flow of 20 mL/min, with a heating rate of 10 °C/min. The procedure of analysis is illustrated in Figure 1.

### 2.4. Characterization of Rubber Vulcanizates

#### 2.4.1. Kinetic of Vulcanization

Vulcanization kinetics of rubber mixes filled with GTR powders were determined with an MDR 2000 oscillating disk rheometer (Alpha Technologies, Hudson, OH, USA), at T = 150, 160 and 170 °C according to PN-ISO 3417. Based on the experimental data, the curing parameters were determined and, namely, optimal vulcanization time (t_90_), vulcanization scorch time (ts_2_), max. (MH), and min. (ML) torque and an increase of torque ΔM = MH − ML were calculated. The conventional cure rate index (CRI) of the rubber compounds studied was calculated according to Equation (1) [28,29]:

(1) $$CRI=\frac{100}{t_{90}-t_{s2}}$$

Based on the vulcametric data, the vulcanization kinetics of the rubber mixes were characterized by the activation energy of vulcanization (E_a_), calculated according to the Arrhenius Formula (2):

(2) $$lnk\left(T\right)=lnA-\frac{E_{a}}{RT}$$

Rate constant (k) was calculated using a nonlinear regression according to the Kamal-Sourour [30] model (3):

(3) $$\frac{d\alpha }{dt}=\frac{1}{M_{H}-M_{L}}\frac{dM}{dt}$$

where: 𝛼(t), M(t)—degree of vulcanization and torque in a given time (t), respectively, enabling determination of the course of vulcanization speed (dα/dt) in function of the degree of vulcanization (𝛼).

#### 2.4.2. Sample Preparation

Samples of rubber vulcanizates were prepared using a hydraulic press under a pressure of 200 bar and a temperature of 160 °C during the optimal time (t_90_), determined rheometrically (PN-ISO 3417).

#### 2.4.3. Degree of Rubber Crosslinking

Crosslinking of rubber vulcanizates was compared calculating their equilibrium swelling in toluene. Samples of ca. 30–50 mg were immersed in a solvent for 72 h. The degree of rubber swelling was calculated applying Formula (4):

(4) $$Q=\frac{\left(\left(m_{s}-m_{n}\right)-\left(m_{0}-m_{n}\right)\right)}{m_{0}-m_{n}}\times 100\%$$

where: Q—equilibrium swelling of rubber, m_s_—weight of a sample after swelling, m_0_–weight of a sample before swelling, m_n_—weight of a filler contained in a GTR together with a carbon black admixed. The measurement error was calculated using the student’s t-test for α = 0.005.

#### 2.4.4. GTR–Polymer Matrix Compatibility

Interactions between the rubber matrix and GTR particles were evaluated by an optical microscope Optatech (Warsaw, Poland), combined with a Leica MZ 6 (Wetzlar, Germany) camera and OptaView (Optatech, Warsaw, Poland) software. The surface of the liquid nitrogen fracture of the elongated samples (100, 150 and 200% obtained with a micrometric screw) was observed under a magnification of 160 times, looking for the GTR particles detaching from polymer matrix.

#### 2.4.5. Mechanical Properties of Rubber Vulcanizates

Rubber vulcanizates containing GTR, subjected to static elongation, were examined with a 1435 universal mechanical testing machine operating with an optical extensiometer (Zwick/Roell, Ulm, Germany), according to PN-ISO 37. Dumb-bell specimens measuring 2.0 ± 0.2 mm thick and 25 mm measuring distance were elongated with 500 mm/min. Six samples per material were tested and the experimental values were averaged. Stress at elongation of 100, 200 and 300 %—SE 100, SE 200 and SE 300 adequately, as well as the elongation at break—Eb were determined. The measurement error was calculated using the student’s t-test for α = 0.005.

#### 2.4.6. Tribological Properties of Rubber Vulcanizates

The friction between rubber vulcanizates and a stainless steel counterface was determined with a steel block-on-rubber ring T-05 tribometer (Research Network “Łukasiewicz”—ITeE, Poland) [31]. The 40 mm diameter rotating rubber ring was sliding against the 10 N normal loaded, stationary polished stainless steel block, with a 0.01 m/s sliding speed. Friction force data were collected during three 1 hr runs and averaged. Additionally, the surfaces of the rubber samples after the test were examined with an Optatech (Warsaw, Poland) optical microscope, operating under a magnification of 40 times.

## 3. Results and Discussions

### Characterization of GTR

**Figure 2:**
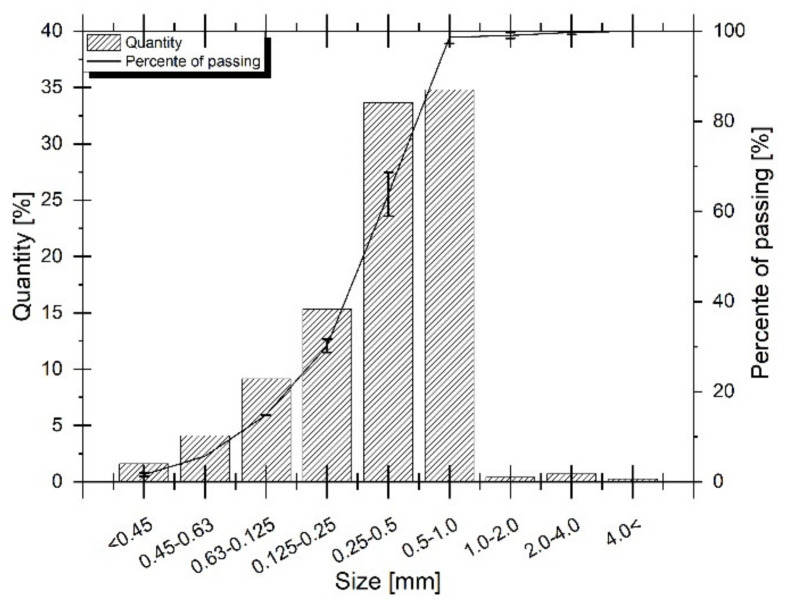
Sieve analysis of the GTR studied.

Sieve analysis of the GTR powder revealed that ca. 98 % of its particles were of a size lower than 1 mm (only this fraction was used as a filler for further studies)—Figure 2. Within this fraction, the biggest population was of a size between 0.25 and 1.0 mm.

**Figure 3:**
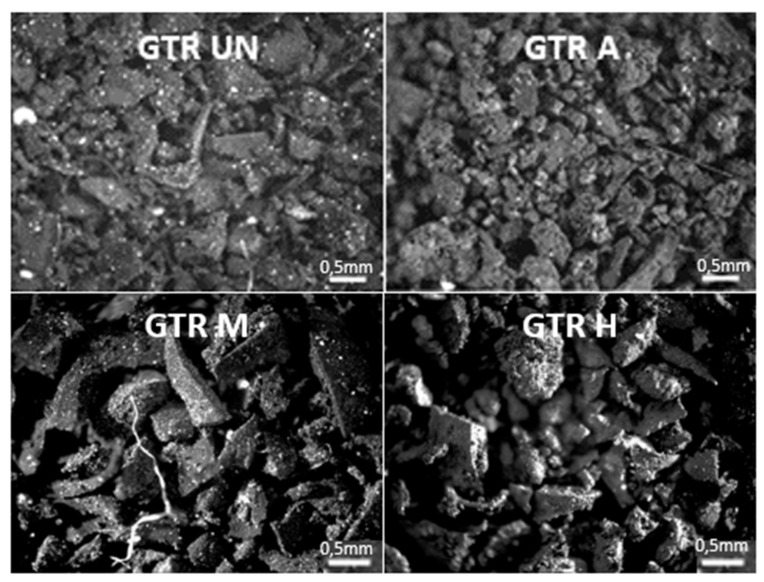
Optical microscope picture of untreated (virgin—GTR UN), sulfuric acid activated (GTR A), subjected to modification (GTR M) or hybrid treatment (GTR H) GTR particles.

Irregularly shaped GTR particles with sharp edges, visible on microscope pictures (Figure 3—GTR UN), suggest that the material was most likely made in a cryogenic process. Moreover, the sharp edges and a relatively low value of the specific surface area of 0.0731 ± 0.0054 m^2^/g indicates this method of grinding [24].

The specific surface area of the ground tire rubber particles treated with sulfuric acid (GTR A) increased ca. 10 times in comparison to the virgin GTR (Table 2), the same as presented by Colom et al. [32]—to 0.6202 ± 0.0224 m^2^/g.

**Figure 4:**
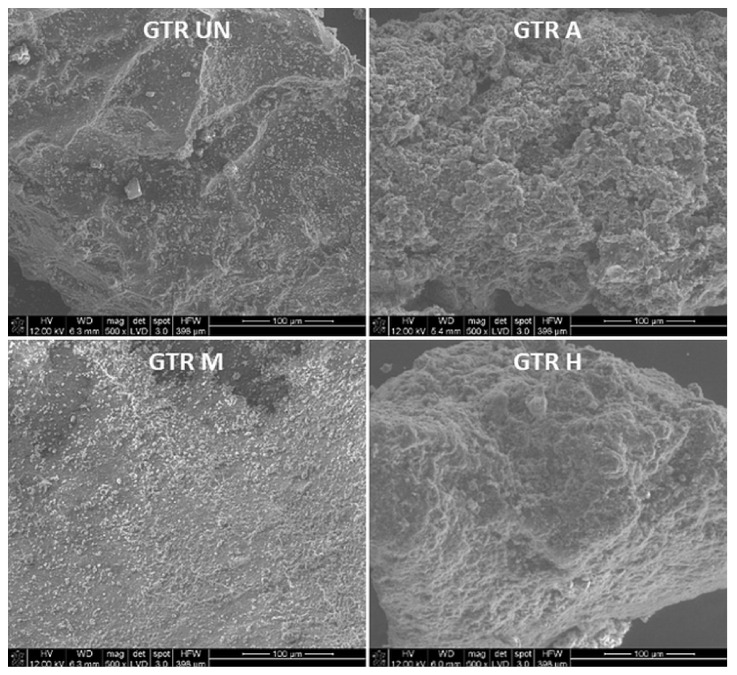
SEM picture of an untreated (GTR UN), subjected to activation (GTR A), modification (GTR M), or hybrid treatment (GTR H) GTR particles’ surface.

The development of SSA of GTR A and GTR H samples can be also seen on the images from an optical (Figure 3) and a scanning electron microscope (Figure 4). The activated GTR particles (GTR A) have more surface cracks and furrows as a result of the treatment, indicating their strong oxidation [33]. The surface of the GTR samples modified applying silane treatment (GTR M) becomes smoothed (Figure 4) and has a BET surface area of 0.0402 ± 0.0070 m^2^/g. Sulfuric acid activating GTR particles makes their surface microcracked and porous, whereas the application of silane makes the particles’ surface smoothed. Nevertheless, the hybrid modification develops the surface of GTR (GTR H) to the highest extent (Figure 4)—the BET surface area reaches a value of 1.2104 ± 0.0129 m^2^/g, which may be due to the penetration between the cracks formed after activation by silane particles and further increasing the surface area. As expected, the size of GTR particles has practically not changed due to the applied modifications.

**Figure 5:**
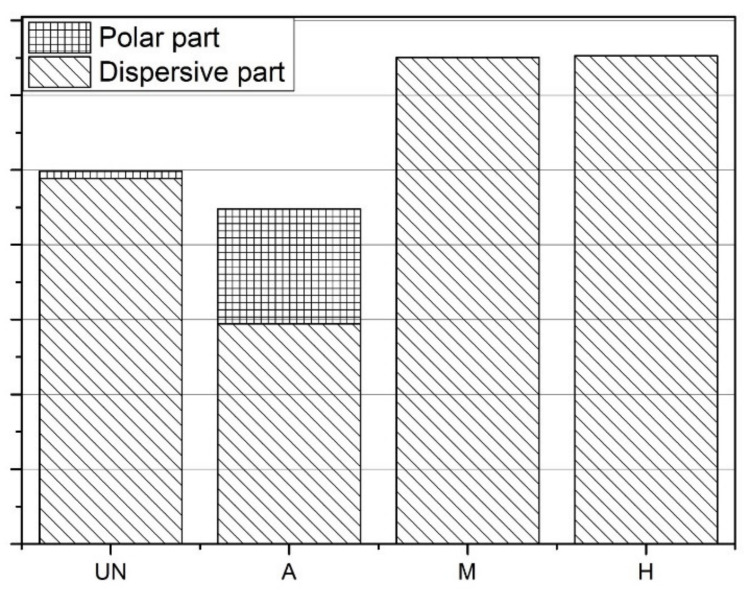
Comparison of the surface free energy of GTRs studied.

Activation of the surface gives a substantial increase in the polar part of surface free energy (Figure 5). It is the effect of using an oxidizing agent and surface development, which enables better adsorption of the solvent during tensiometric testing. Each use of silane causes a decrease of the polar part of SFE to 0. This may be due to the reaction between the polar groups on the surface and the added agent. Nevertheless, it results in the highest value of the surface free energy.

The range of changes concerning the specific surface area of GTRs accompanying the modification of their particles, due to their low absolute SSA values in comparison to active fillers, cannot be responsible for significant changes in the mechanical properties of rubber vulcanizates filled with ground tire rubber. The changes should rather be the result of the chemical modification of the GTRs’ surface, enhancing interphase interactions with the rubber matrix. Apart from this, the modification is also likely to affect the curing process due to the possibility of taking part in rubber crosslinking [34,35]. Thermogravimetric analysis of GTRs revealed the biggest changes in composition after acid activation (GTR A). Sulphuric acid can react with rubber, facilitating water adsorption on the particles’ surface [36], being released during heating, and the decrease of rubber content in GTR. The former is the most visible for hybrid modification (GTR H) and influences the amount of caoutchouc content in the GTR studied—Table 3—used for the recalculation of the rubber mixes’ composition.

**Figure 6:**
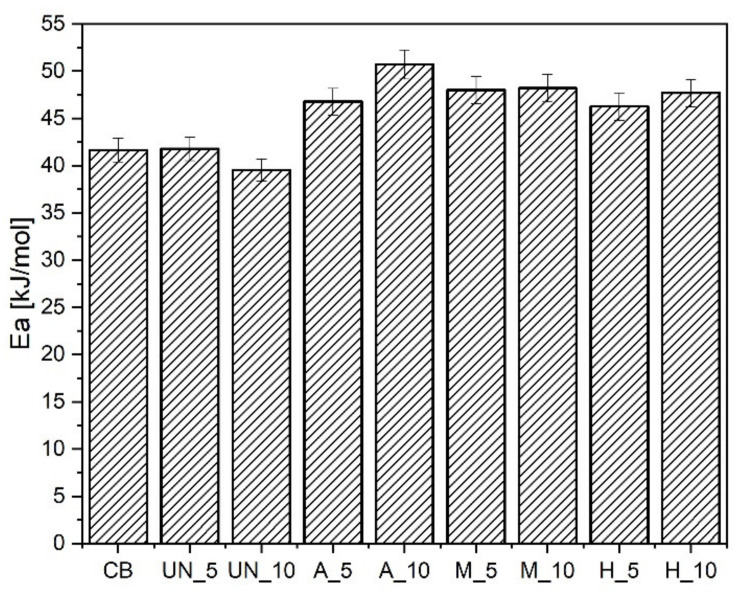
Activation energy of vulcanization for the samples containing GTR.

**Figure 7:**
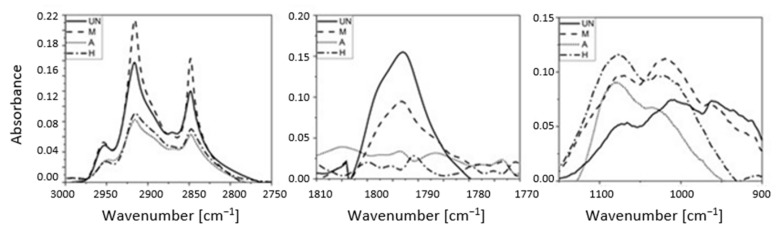
FT-IR spectra for GTRs’ particles.

The addition of a small amount of unmodified GTR does not affect the activation energy (Ea) of the vulcanization process (Figure 6). Increasing its addition (UN10) causes a slight decrease in Ea, which may originate from a lower amount of raw polymer in the mix. The addition of an acid-activated additive increases the activation energy of vulcanization, which is likely to be the result of its surface acidification. The effect is the most visible for a A10 sample. However, changes to the surface composition of modified GTRs are the most visible for silanized (M), or the powder modified in the hybrid way (H). FTIR spectra (Figure 7) and EDS analysis (Table 4) demonstrate a significant difference in the intensity of absorbance bands originated from hydroxyl groups (3440 cm^−1^), methyl groups (2960 cm^−1^), methylene groups (2920 and 2850 cm^−1^), as well as C = O groups (1720 cm^−1^), -C = C- (1640 cm^−1^), C–O stretching (1100 cm^−1^) and Si-O-Si groups (1010 cm^−1^) for the former, and the values of oxygen signal for the latter analysis.

Untreated GTR showed signals at 2960, 2920, and 2850 cm^−1^, which can be ascribed to the C-H stretching. After silanization, these bands (especially the last two) increased in intensity, whereas when GTR was subjected to activation with sulfuric acid or hybrid treatment, these bands decreased in intensity. Simultaneously, new absorption bands were detected, namely -C=C- at 1640 cm^−1^ and carbonyl groups (-C = O) at 1720 cm^−1^. The presence of these carbonyl groups is frequently correlated with hydroxyl groups (-OH). The characteristic broad absorption band at 3440 cm^−1^, corresponding to the stretching vibration of the -OH group, resulted from the oxidation processes, showing a more intense signal when GTR was treated with H_2_SO_4_. A strong band corresponding to C–O stretching was also observed at 1100 cm^−1^ for the GTR activated with sulfuric acid. Alternatively, in the case of the chemical treatment with H_2_SO_4_, the two peaks observed in the 1000–1200 cm^−1^ region could also indicate the O = S = O stretching [37]. These new chemical groups grafted onto the surface of GTR are not able to improve the interfacial adhesion with SBR matrix; however, their polarity facilitates interactions with silane and their reinforcing efficiency [14].

**Figure 8:**
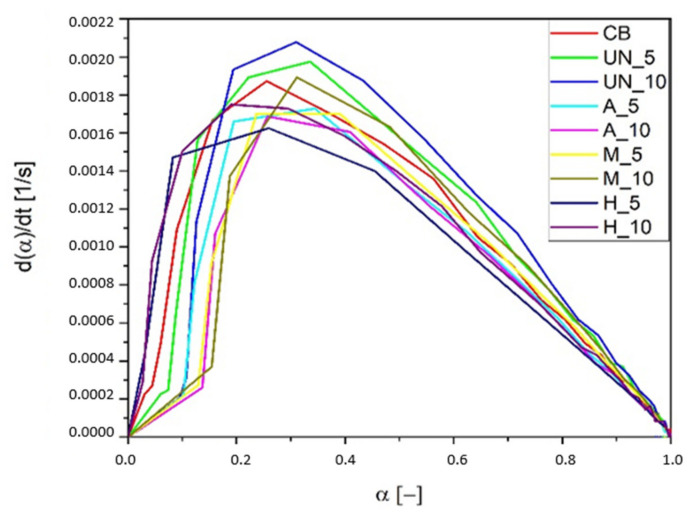
Vulcanization speed of the rubber mixes containing various amounts of GTRs modified in different ways in function of the vulcanization degree.

Acid activation (A) of GTR particles characterize themselves by the highest activation energy of vulcanization, when admixed to SBR, within the ground tire rubbers studied. During the second stage of the hybrid modification (H), silanization, the surface acidity decreases, which results in the lowering of the energy required to initiate the crosslinking process, similar to silane treatment (M). GTR particles covered by silane also introduce an additional amount of sulfur, which is confirmed by FTIR spectra (Figure 7—two peaks in the region 1100–1200 cm^−1^) and EDS data (Table 4). Due to all the differences visible in the materials, it can be concluded that, apart from the physical modification produced by GTR addition to rubber mixes, its surface composition is also important from the point of view of the crosslinking of rubber mixes and the properties of the rubber vulcanizates. Parameters of vulcanization—Table 5—determined from the kinetics of the vulcanization of GTR-filled SBR show that all samples containing the rubber powders have lower t_s2_ and t_90_ values compared to the material filled with carbon black (CB). The addition of GTR (UN) causes an increase in CRI, which results from the fact that less caoutchouc is subjected to crosslinking. Silanization of GTR particles introduces thiol groups, which should also contribute to crosslinking. However, possible influence of residues of the crosslinking system on the surface of ground tire rubber particles is negligible due to their low SSA value. Acid activation (A) reduces the CRI value, due to the negative effect of acidic pH (from unwashed residues) on sulfur crosslinking. The silane modification (M) does not have a significant effect on the CRI, while in the case of hybrid samples (H), a significant decrease in CRI is visible, which may be due to the presence of thiol groups on the previously activated GTR surface (which is likely to some extent to participate in crosslinking [34,35]). The above relationships are also visible in the dα/dt diagram (Figure 8).

**Figure 9:**
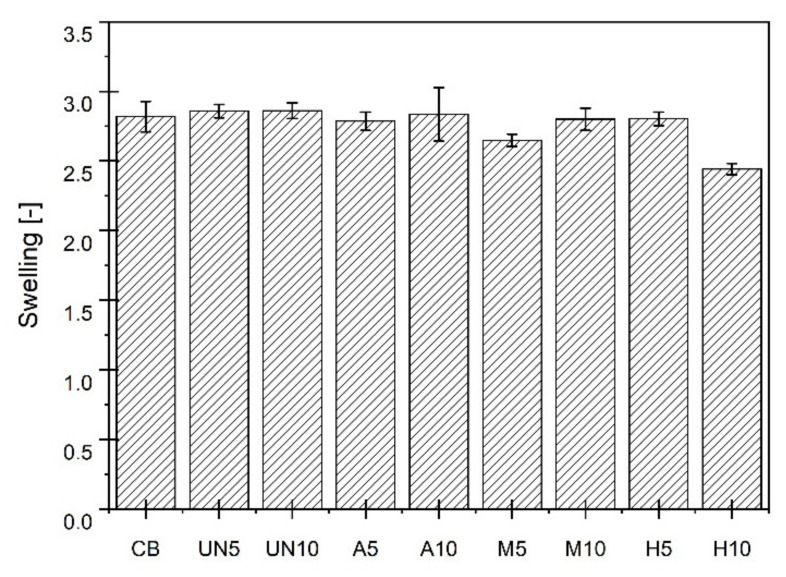
Equilibrium swelling of the rubber vulcanizates studied in toluene.

The differences observed are clearly the result of a lower amount of raw rubber in mixes. However, they can also originate either from some residues of the crosslinking system left on the surface of GTR particles, or they are an effect of silane treatment. As expected, the introduction of GTRs to rubber mixes, whether virgin or modified, results in worse properties in comparison to rubber filled with carbon black. Supreme effects were observed for the samples containing 5 wt. parts of rubber from silanized GTR (M5) and 10 wt. parts of rubber from GTR subjected to hybrid treatment (H10), probably because of their higher filler loading (Table 1) or higher crosslink density (Figure 9), respectively, in comparison to the other materials studied.

**Figure 10:**
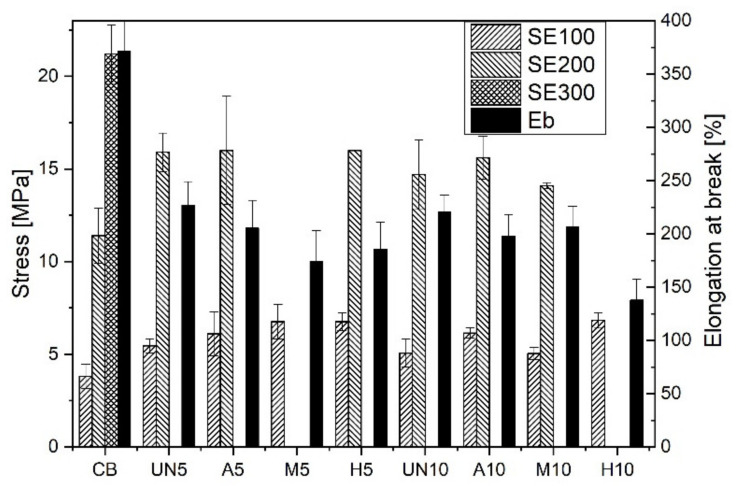
Mechanical properties under elongation of the rubber vulcanizates studied. Stress under elongations at 100% (SE100), 200% (SE200), 300% (SE300) and the elongation at break (Eb).

Results of basic mechanical tests for the rubber vulcanizates (Figure 10) are in agreement with the previous observations concerning the changes to their equilibrium swelling. The addition of GTR powder makes the stiffness of rubber vulcanizates increase, as reported similarly by Colom [32]. Their elongation at break seldom exceeds 200%.

**Figure 11:**
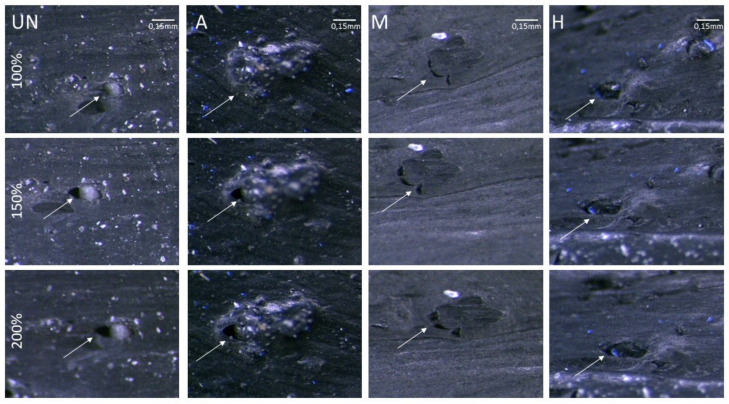
Microscopic picture of fractured samples containing untreated GTR (UN5) and GTR after acid activation (A5), silane modification (M5), and hybrid treatment (H5). Arrows indicate on the holes created between GTR particles and polymer matrix.

Proposed methods for the surface modification of GTR do not lead to any significant changes in the mechanical properties of rubber vulcanizates with their addition when compared to SBR filled with the virgin ground tire rubber powder. The viscoelastic properties of the materials could be studied more deeply, e.g., by applying the analysis proposed recently by Cacopardo et al. [38]. The highest differences can again be subscribed to the samples M5 and H10, being stiffer (the highest SE 100 values and Eb ones do not reach even 200%) in comparison to the rest of the rubber vulcanizates studied. Arguably, it could also be an effect of increased interactions between GTR and the rubber matrix, being the consequence of the presence of silane thiol groups on the particles’ surface. It could be also the reason for their lowest equilibrium swelling values compared to the other compounds studied (Figure 9). Microscopic pictures of rubber under elongation (Figure 11) illustrate well the different interactions between the rubber matrix and GTR particles. The figure presents images of the materials where 5 wt. parts of rubber were replaced by rubber coming from GTR. Observations are analogous for the samples where 10 wt. parts of SBR were substituted with GTR rubber. In the fracture of SBR filled with untreated GTR, it can be seen that GTR particles do not elongate during stretching. A distinct hole appears between a fine GTR particle and the polymer matrix, which can be due to the very smooth GTR surface and the lack of interphase interactions of a chemical nature. A similar effect is visible in the case of the GTR subjected to acid activation; however, due to the significant increase in the surface area, the hole is much smaller than observed for the unmodified material. The use of GTR after silanization or hybrid treatment significantly reduces the hole size formed during stretching; furthermore, the GTR particles are connected to the matrix and stretch analogously to the polymer matrix.

**Figure 12:**
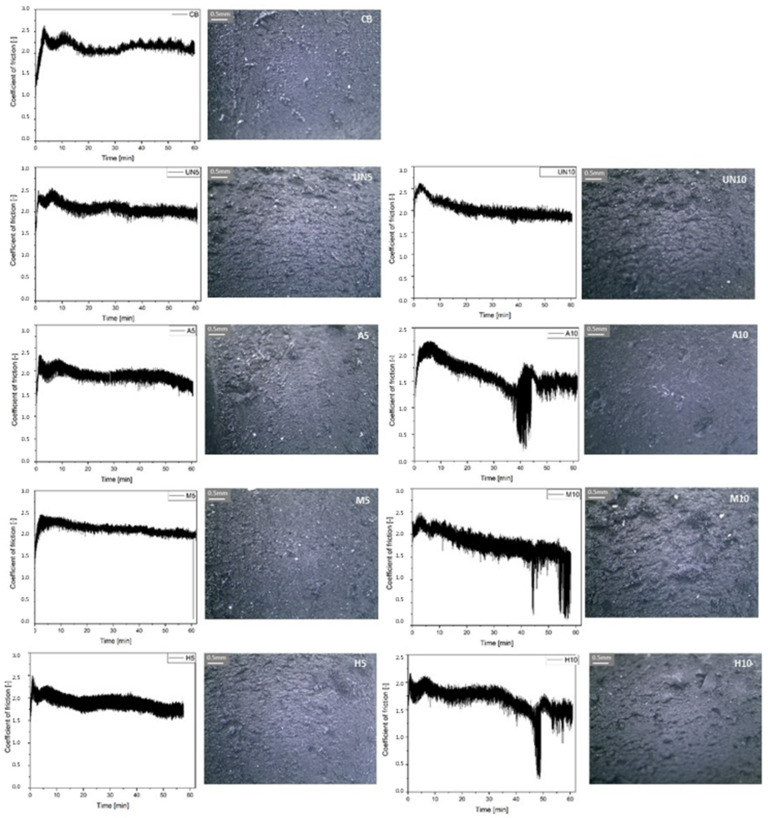
Friction coefficient in function of time for the rubber vulcanizates studied and optical microscope pictures of their surface after tribological tests.

Character of interphase as well as interface interactions significantly affect the friction and wear of filled rubber vulcanizates [39]. They influence the stiffness and hysteretical components of friction and determine the abrasion of the material. Generally, samples in which 5 wt. parts of rubber were replaced by rubber coming from GTR are comparable to the rubber filled with CB, no matter the GTR modification (Figure 12). The coefficient of friction is only lower for the sample H5, which may be caused by an excess of unreacted silane lubrication [40]. Abrasive wear of the samples where 5 wt. parts of SBR were replaced by rubber from GTR is lower in comparison to the samples with 10 wt. part substitution. This is because the probability of GTR particle separation from the worn surface is much higher for the latter, which manifests itself in high damage to the surface, accompanied by a significant drop in friction coefficient. Destruction can be visible in the vulcanizates containing the modified material. In the sample containing unmodified GTR (UN10), the course of friction remains practically the same as detected for UN5. SBR modified by a higher amount of acid-activated GTR (A10) exhibited the lowest resistance to abrasive destruction (after ca. 40 min of the test), followed by the rubber filled with silanized GTR (M10) and GTR treated in a hybrid way (H10), for which the destruction occurs after ca. 44 min and 49 min, respectively, probably being delayed thanks to the strongest GTR–polymer matrix interphase interactions. The addition of the modified GTR results in a significant lowering of friction in comparison to rubber vulcanizates containing virgin GTR (UN10), which can be explained by their stronger interactions with the SBR matrix. The microscopic pictures of wear traces confirm the greatest traces of wear are present in the case of the UN10 rubber vulcanizate sample. Furrows and hills visible on the surface are also high in the case of M10. This time, however, they probably originate from wear debris contributing to the morphology due to the enhanced wear of SBR plastified by an excess of silane that cannot be used for the surface modification of GTR particles. 

## 4. Conclusions

The paper compares original methods of GTR modification from the point of view of their application to compatibilize the waste filler with rubber matrices. Insufficient interactions between GTR particles and the rubber matrix make mechanical properties of vulcanizates containing GTR deteriorate. The results presented in Figure 10 demonstrate that even without further grinding, the GTR particles of such a big size (the particle size of GTRs strongly influence the mechanical properties of SBR vulcanizates [33]) could be used as a filler in rubber vulcanizates in undemanding applications. It seems likely that it could replace, to some extent, carbon black in rubber mix compositions, favoring the management of rubber waste. However, generally, the addition of GTR powder adversely affects the kinetics of vulcanization and the mechanical properties of rubber vulcanizates, which is reflected in the decreased scorch time and lower tensile strength and the elongation at break of the vulcanizates containing GTRs [41]. The reason for that can be subscribed to the low specific surface area and general incompatibility between GTR particles and rubber matrices. Slightly higher crosslink densities, resulting from the lower amount of raw rubber in the mixes and/or residues of crosslinking agents on the surface of the powder particles, clearly contribute to this effect, but cannot be the main reason responsible for it. Considerable effects, obtained by sulphuric acid activation of the surface of GTR powder, resulting in changes to its surface microroughness (due to oxidative shrinkage followed by microcracking) and chemical composition, open the way to effective hybrid modification of the powder by silanization. It results in improved compatibilization of GTR particles with rubber matrices, additionally enhancing their crosslinking due to the silane contribution. The effect of GTR modification from the point of view of its application as a secondary filler of SBR allows for applications up to ca. 25 phr of GTR in a rubber mix, still maintaining reasonable mechanical and good tribological properties of the vulcanizates. 

## Figures and Tables

**Table 1:** Composition of the rubber mixes studied (phr).

	CB	5 Parts of Rubber from GTR				10 Parts of Rubber from GTR			
		UN	A	M	H	UN	A	M	H
SBR Ker 1500	100.0	95.0				90.0			
Stearic acid	1.0								
Zinc oxide	3.0								
CBS	1.0								
Sulfur	2.0								
Carbon Black N330	50.0	44.0	44.2	44.0	43.3	38.0	38.4	38.1	36.6
GTR	-	11.5	12.4	11.6	12.9	23.0	24.8	23.2	25.7

**Table 2:** Specific surface area (SSA) of the GTRs studied as determined by BET.

GTR Sample	SSA [m^2^/g]
UN	0.0731 ± 0.0075
A	0.6202 ± 0.0310
M	0.0402 ± 0.0097
H	1.2104 ± 0.0179

**Table 3:** GTRs phase composition determined by thermogravimetric analysis.

Phase	GTR			
	UN	A	M	H
Low molecular weight organic substances/water, wt. %	4.5	13.0	5.5	9.0
Rubber, wt. %	43.4	40.2	43.1	38.9
Carbon black and mineral substances, wt. %	52.1	46.8	51.4	52.1

**Table 4:** Composition of GTRs studied by EDS (limited to the most important elements).

Composition [at. %]	GTR			
	UN	A	M	H
Carbon	90.24	93.57	88.21	91.07
Oxygen	4.87	3.95	7.04	5.18
Silicon	0.49	0.31	1.93	0.74
Sulfur	1.01	1.06	1.33	1.66

**Table 5:** Parameters of vulcanization of the GTR-containing rubber mixes studied.

Parameter Sample	M_L_ [dNm]	M_H_ [dNm]	∆M	t_s2_ [min]	t_90_ [min]	CRI [%/min]
CB	2.6	20.6	18.0	4.6	18.2	8.6
UN_5	2.6	19.1	16.5	4.4	14.5	9.9
UN_10	2.7	18.4	15.7	4.1	13.3	10.8
A_5	2.7	20.7	18.0	4.3	15.8	8.7
A_10	3.0	20.8	17.8	4.0	15.7	8.6
M_5	2.7	19.2	16.5	4.3	14.9	9.4
M_10	3.1	17.6	14.5	3.9	13.4	10.5
H_5	2.6	20.7	18.1	4.2	16.2	7.2
H_10	2.8	19.5	16.7	4.2	16.5	8.1
